# The Use of Artificial Intelligence to Improve Readability and Quality of Informed Consent Forms in Plastic and Reconstructive Surgery

**DOI:** 10.1055/a-2870-8035

**Published:** 2026-07-07

**Authors:** Grace S. Ryu, Grace Hwang, Charles C. Lee, Timothy S. Johnson

**Affiliations:** 1 12310 College of Medicine, Penn State Milton S. Hershey Medical CenterHershey PennsylvaniaUnited States; 2 Division of Plastic Surgery Department of Surgery 328945 Penn State Milton S. Hershey Medical CenterHershey PennsylvaniaUnited States

**Keywords:** plastic and reconstructive surgery, consent forms, artificial intelligence, readability, quality

## Abstract

**Background:**

The readability and length of informed consent forms have been debated for over three decades. Despite national guidelines promoting patient-friendly language, many forms fall short. This study compares the readability and content quality of informed consents from the American Society of Plastic Surgeons (ASPS) and ChatGPT for common plastic and reconstructive surgery (PRS) procedures.

**Methods:**

ASPS consents for seven common PRS procedures were obtained online. ChatGPT was then queried to create equivalent consents. Readability was assessed using the Flesch Reading Ease Score, Flesch–Kincaid Grade Level, and Lexical Density. Quality was graded using a modified National Quality Forum-based scale across domains: Procedure description, clinical rationale, patient-oriented benefit, quantitative and qualitative risks, and alternatives. Scores were compared between ASPS and ChatGPT consent forms.

**Results:**

ASPS versus ChatGPT conversion yielded mean Flesch scores of 30.09 versus 46.18 ( *p* = 0.00003), grade levels of 13.59 versus 10.53 ( *p* = 0.000004), and lexical densities of 56.7% versus 46.6% ( *p* = 0.00008). Word count decreased by 81% after conversion. ASPS consents exceeded ChatGPT quality in three of seven procedures (by 12.8 percentage points on average), while ChatGPT exceeded ASPS in four of seven (by 25 points on average). Average quality scores were not significantly different (64.83% vs. 73.63%, *p* = 0.27).

**Conclusion:**

ChatGPT improved the readability and brevity of PRS consent forms while maintaining accuracy. Readily available artificial intelligence (AI) tools may enhance descriptions of procedures, rationale, patient benefits, and quantitative risks, offering a practical tool to aid in patient–provider communication.

## Introduction

According to the American Medical Association's Code of Medical Ethics, informed consent is central to patients' decision-making process regarding their care. 1 A patient-friendly informed consent form should be detailed, readable, and brief, while including a full overview of the clinical condition, proposed and alternative interventions, and the associated risks and benefits. 1 2

Given the legal, ethical, and administrative purposes that informed consents serve, the readability, length, and quality of these forms have been under review for over three decades. 2 3 To optimize patient comprehension, informed consents should be written at the fourth- to sixth-grade reading level. 4 5 However, a study evaluating informed consents from 104 U.S. hospitals found that, on average, text was written at the 11th-grade reading level. 6 Additionally, informed consents in plastic and reconstructive surgery (PRS) have been evaluated to be focused on risk disclosures using complex medical terminology with efforts to mitigate surgeon liability, rather than explanation of risks for the patient's understanding. 6 7 Poor readability of forms can negatively impact an individual's ability to weigh treatment options and make informed medical decisions. 8 9

Amending current informed consents to meet patient-centered standards would require immense resources from institutional administration and clinical staff. 10 Large language models, such as ChatGPT, can quickly generate large volumes of text about surgical procedures. 11 12 13 Recent research found that artificial intelligence (AI) can generate informed consent forms for common surgical procedures, including Mohs micrographic surgery and laparoscopic appendectomy, with optimized readability while preserving medical and legal content. 14 Although ChatGPT has been used to generate plastic surgery operative notes, no studies to date have generated and assessed AI-generated informed consent forms in PRS. 15 The purpose of this study was to compare the readability and quality of content between informed consents by the American Society of Plastic Surgeons (ASPS) and by ChatGPT for procedures commonly performed in the field of PRS.

## Methods

Informed consents created by ASPS were obtained online for seven notable PRS procedures: Breast reconstruction with deep inferior epigastric perforator (DIEP) flap, breast reconstruction with tissue expander, breast reduction mammoplasty, abdominoplasty, augmentation mammoplasty, skin lesion removals, and functional panniculectomy. ChatGPT v3.5 was queried to “generate a patient-friendly informed consent form” for each procedure. Once these forms were generated, ChatGPT was not queried again to produce revised consent forms. Readability and content materials were assessed using the Flesch Reading Ease Score, Flesch–Kincaid Reading level, word count, and lexical density. The Flesch Reading Ease Score and Flesch–Kincaid Reading level were calculated by ChatGPT, and word count and lexical density were calculated using Online Utility, which is a publicly available online interface in which users can insert text to determine text content, including word count, lexical density, and readability. This interface can be found at https://www.online-utility.org/text/analyzer.jsp .

Quality was assessed by two independent authors of this manuscript, G.S.R. and G.H., using a modified National Quality Forum (NQF) scale. 16 The NQF scale grades the quality of informed consents in several domains, including descriptions of the procedure, clinical rationale, patient-oriented benefits, quantitative risks, qualitative risks, and alternatives. The NQF quality score is calculated out of 20. For this study, we modified the NQF scale to be calculated out of a total of 13, with 13 representing the highest quality (see Appendix [available in the online version only]). We omitted question 1a (If [description of procedure is provided], is it typed?) and question 2a (If [description of how procedure is done], is it typed?) because all informed consents evaluated in this study are typed. We also omitted question 8, which evaluated whether informed consent was provided to patients prior to the day of the procedure, because this study did not provide consent forms to real patients.

Prior to review, the two reviewers' (one resident physician and one attending physician, both native English speakers) scores were discussed, and if a domain had a difference of two or more points, further discussion would need to take place to maintain the integrity of interobserver reliability. Intraclass correlation was calculated to assess interobserver reliability.

Individual domain scores and total quality scores were compared across ASPS and ChatGPT-generated consents for each included procedure using a *t* -test. Analyses were done using Google Sheets (Google LLC, Mountain View, CA; accessed January 2025). *p* -Values <0.05 were considered statistically significant.

## Results

The mean Flesch Reading Ease scores for ASPS informed consent forms before and after ChatGPT conversion were 30.09 (standard deviation [SD] 1.55) and 46.18 (SD 5.98, *p* < 0.001), the Flesch–Kincaid grade levels were 13.59 (SD 0.18) and 10.53 (SD 0.94, *p* < 0.001); and the lexical densities were 56.7 (SD 0.54) and 46.6 (SD 4.23, *p* < 0.001), respectively ( Table 1 ). The average word count for ASPS consent forms was 5256.57 (SD 1,232.67) and 958.71 (SD 152.04) for ChatGPT-generated consent forms ( Table 1 and Fig. 1 ). There was an 81% reduction in word count after conversion. A sample of the original ASPS informed consent form and the ChatGPT-generated text is shown in Table 2 .

**Fig. 1:**
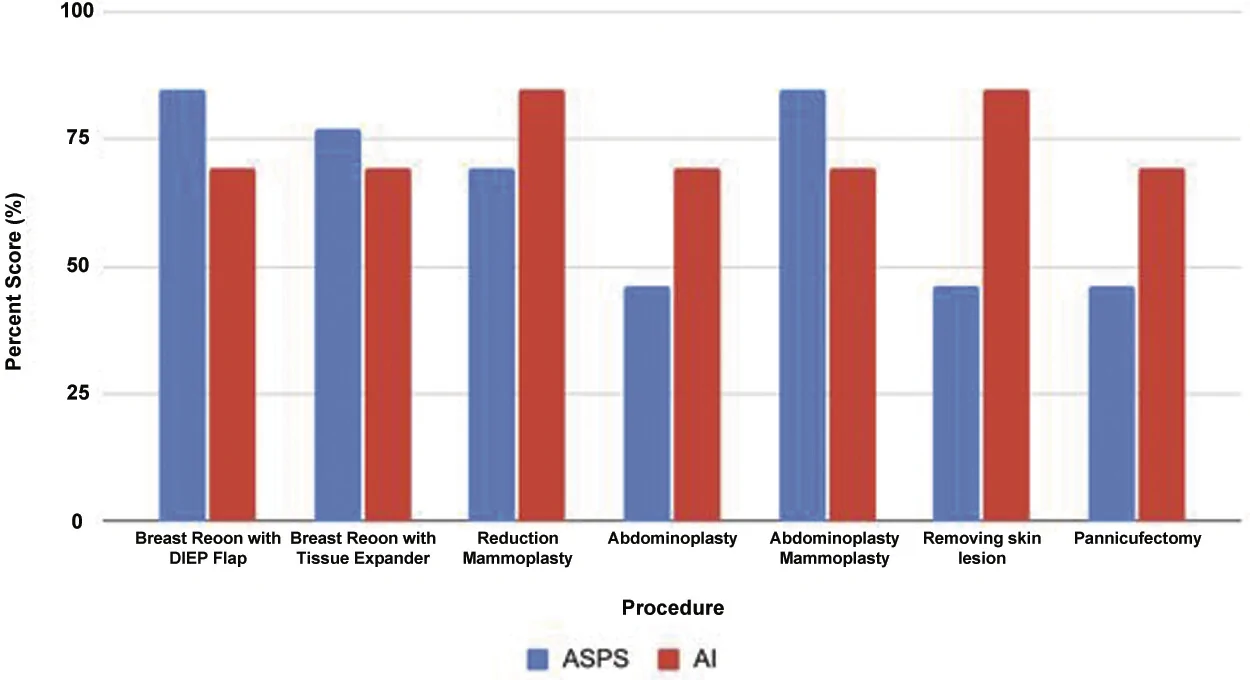
Overall quality scores. AI, artificial intelligence; ASPS, American Society of Plastic Surgeons; DIEP flap, deep inferior epigastric perforator flap.

**Table 1:** Summary of readability assessment

	ASPS forms	ChatGPT-generated consent forms	*p* -Value
Flesch Reading Ease	30.09	46.18	0.00003
Flesch–Kincaid	13.59	10.53	0.000004
Word count	5,256.57	958.71	0.000002
Lexical density	56.7	46.55	0.00008

Abbreviation: ASPS, American Society of Plastic Surgeons.

**Table 2:** Samples of text from the American Society of Plastic Surgeons informed consent forms converted by artificial intelligence

Original text	Converted text
**Risks of Breast Reconstruction with Tissue Expander** a Every surgical procedure involves a certain amount of risk, and it is important that you understand the risks involved with breast reconstruction with a tissue expander. In addition, every procedure has limitations. An individual's choice to undergo a surgical procedure is based on the comparison of the risk to the potential benefit. While the majority of women do not experience these complications, you should discuss each of them with your plastic surgeon to make sure you understand the risks, potential complications, and consequences of breast reconstruction with a tissue expander. Problems associated with breast implants and tissue expanders can be inherent to this type of implanted medical device or relate to complications of a surgical procedure. Patients considering surgery that involves breast implants and tissue expanders should review additional advisory information regarding this subject. Additional information concerning breast implants and tissue expanders may be obtained from the FDA, package-insert sheets supplied by the device manufacturer, or other information pamphlets required by individual state laws. While every patient experiences her own individual risks and benefits following tissue expander breast reconstruction, clinical data suggest that most women will be satisfied with the outcome of surgery despite the occurrence of problems inherent with breast implant and tissue expander surgery. **Inherent Risks of Saline-Filled Breast Implants/Tissue Expanders** **Tissue expanders/implants** : Tissue expanders and implants, similar to other medical devices, can fail. Tissue expanders and implants can break or leak. When a saline-filled implant or tissue expander ruptures, the body absorbs the saline material, but the shell material remains. Rupture can occur because of an injury, from no apparent cause (silent rupture), or during mammography. It is possible to damage a tissue expander or implant at the time of surgery or subsequently with a needle during the insertion of saline into the device. Damaged, leaking, or broken tissue expanders and implants cannot be repaired and require replacement or removal. Breast implants and tissue expanders can wear out; they are not guaranteed to last a lifetime, and future surgery may be required to replace one or both implants. An MRI (magnetic resonance imaging) study may be necessary to evaluate the possibility of device rupture or deflation, but it may not be 100% accurate in diagnosing integrity. Saline-filled breast implants may not have the same contour or feel as silicone-filled breast implants. The shape of your breasts after surgery depends on many factors, such as your skin thickness, position, placement of the implants or expanders, and technique. You should discuss with your surgeon the possibility of a different and less-than-desirable contour shape, as well as the feel of your result. **Capsular contracture** : Scar tissue, which forms internally around the tissue expander, can tighten and make the breast round, firm, and possibly painful. Excessive firmness of the breasts can occur soon after surgery or years later. The occurrence of symptomatic capsular contracture is not predictable. The incidence of symptomatic capsular contracture can be expected to increase over time. Capsular contracture may occur on one side, both sides, or not at all. It is more common with tissue expander placement in front of the chest muscle layer. Treating capsular contracture may require surgery, tissue expander replacement, or tissue expander removal. Capsular contracture may re-occur after surgical procedures to treat this condition. **Implant extrusion/tissue necrosis** : Lack of adequate tissue coverage or infection may result in exposure and extrusion of the tissue expander or implant through the skin. Tissue breakdown (necrosis) has been reported with the use of steroid drugs, after chemotherapy/radiation to breast tissue, due to smoking, microwave diathermy, and excessive heat or cold therapy. In some cases, incision sites fail to heal normally. A tissue expander or implant may become visible at the surface of the breast as a result of the device pushing through layers of skin. If tissue breakdown occurs and the tissue expander or implant becomes exposed, removal may be necessary. Permanent scar deformity may occur. **Change in nipple and skin sensation** : Breast reconstruction cannot restore normal sensation to the breast or nipple. Changes in sensation may affect sexual response or the ability to breastfeed a baby. **Skin wrinkling and rippling** : Visible and palpable wrinkling of implants or tissue expanders and breast skin can occur. Some wrinkling is normal and expected. This may be more pronounced in patients who have saline-filled implants with textured surfaces or thin tissue. It may be possible to feel the tissue expander fill valve. Some patients may find palpable valve and wrinkles cosmetically undesirable. Palpable valve, wrinkling, and/or folds may be confused with palpable tumors, and questionable cases must be investigated. **Calcification** : Calcium deposits can form in the scar tissue surrounding the tissue expander and may cause pain, firmness, and be visible on mammography. These deposits must be identified as different from calcium deposits, which are a sign of breast cancer. Should this occur, additional surgery may be necessary to remove and examine calcifications. **Chest wall irregularities** : Chest wall irregularities have been reported secondary to the use of tissue expanders and breast implants. Residual skin irregularities at the ends of the incisions or “dog ears” are always a possibility when there is excessive redundant skin. This may improve with time, or it can be surgically corrected. **Implant displacement and tissue stretching** : Displacement, rotation, or migration of a breast implant or tissue expander may occur from its initial placement and can be accompanied by discomfort and/or distortion in breast shape (visible rippling of the skin). Unusual techniques of placement may increase the risk of displacement or migration. Additional surgery may be necessary to attempt to correct this problem. It may not be possible to resolve this problem once it has occurred. **Surface contamination** : Skin oil, lint from surgical drapes, or talc may become deposited on the surface of the tissue expander or implant at the time of insertion. The consequences of this are unknown. **Unusual activities and occupations** : Activities and occupations that have the potential for trauma to the breast could potentially break or damage a tissue expander or implant, or cause bleeding/seroma. **MRI examination during the expansion period** : Most of the expanders have a magnet at the injection site to allow for easier localization of the injection port during the expansion period. MRI uses very strong magnetic fields, which may cause movement, heating, or dislocation of the expander. For this reason, patients with a breast expander in place should not undergo MRI imaging until the expander is removed and replaced with an implant. **Use of acellular dermal matrix** : In order to place the expander in the right position and maintain that position, your plastic surgeon may choose to use biological materials. Most commonly, these materials are derived from human cadaver skin or pig skin. These materials are generally processed and do not carry any viable cells. You should ask your surgeon about these materials. They assist in contouring the pocket around the implant, provide additional cover to an implant, and become populated with your cells, becoming similar to your own tissue. These acellular products may produce fluid and require drains for a prolonged period of time.	**Risks and complications** b All surgeries carry some risk. Below are the potential risks and complications associated with tissue expander reconstruction: **General Risks of Surgery** ● Infection: An infection can develop at the surgical site and may require antibiotics or, in some cases, removal of the expander. ● Bleeding: You may experience bleeding during or after the surgery. ● Scarring: Any surgery will leave scars, though they tend to fade over time. ● Anesthesia risks: Complications can arise from anesthesia, including allergic reactions and breathing difficulties. **Specific Risks of Tissue Expander Surgery** ** Expander rupture or leaking** : The tissue expander could rupture or leak, requiring additional surgery to fix or replace it. ** Pain or discomfort** : Some patients may feel tightness or discomfort as the expander stretches the skin. ** Infection around the expander** : In some cases, an infection may occur around the expander, requiring it to be removed. ** Capsular contracture** : Scar tissue can form around the expander or implant, causing the breast to feel hard. This may require surgery to correct. ** Asymmetry** : Your breasts may not match perfectly in size, shape, or position, which could require additional procedures. **Skin problems** : The skin over the expander could become thin, or the expander might shift out of place.

Abbreviation: ASPS, American Society of Plastic Surgeons.

a Sample of an informed consent form regarding risks for breast reconstruction with tissue expander from ASPS.

b Sample of an AI-generated informed consent form regarding risks for breast reconstruction with a tissue expander.

The intraclass correlation coefficient for quality scores was calculated to be 0.91 ( *p* < 0.001). On average, quality scores were 64.83% (SD 18.24) and 73.62% (SD 7.50), respectively, for ASPS and ChatGPT consent forms ( *p* = 0. 27; Table 3 and Fig. 2 ). Every informed consent fulfilled minimum criteria for describing the procedure beyond the medical name and providing a qualitative probability for any procedure-specific risk and providing alternatives. However, all informed consents failed to provide a quantitative probability for procedure-specific risks. A two-tailed, paired *t* -test was performed, and there was no significant difference in the average score for description of how the procedure will be performed ( *p* = 0.60) and provision of patient-oriented benefit ( *p* = 0.69) between ASPS informed consents versus ChatGPT informed consents. There was a statistically significant difference in the average score for provision of a condition-specific clinical rationale for the procedure ( *p* = 0.03). For this domain, ASPS informed consents had an average score of 1.43/2. ChatGPT informed consents had an average score of 2/2. See Table 4 for mean quality scores for each domain of the modified NQF scale.

**Fig. 2:**
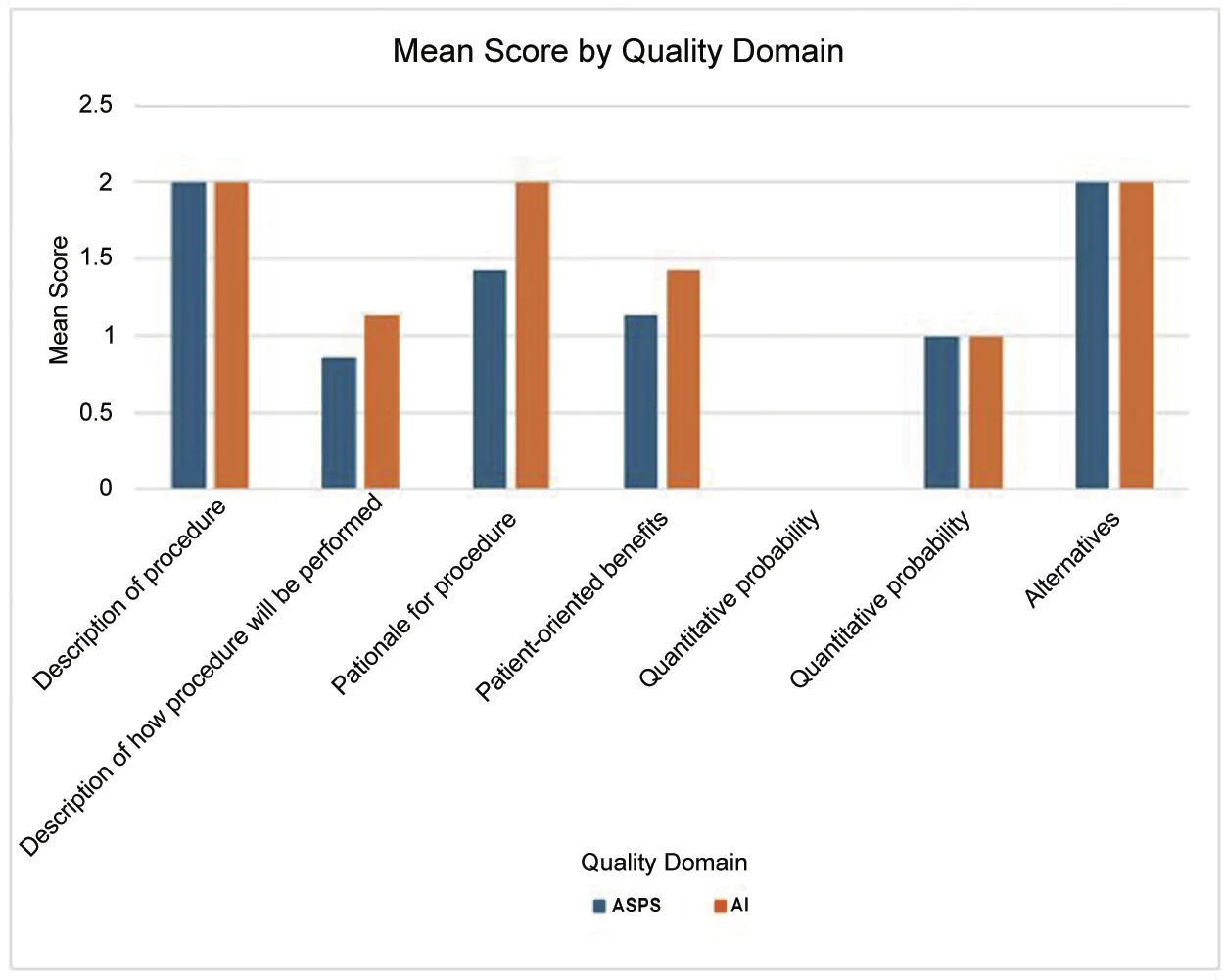
Mean quality scores by domain assessed by National Quality Forum (NQF). AI, artificial intelligence; ASPS, American Society of Plastic Surgeons.

**Table 3:** Quality scores assessed by National Quality Forum

	ASPS (%)	AI (%)	*p* -Value
Breast recon with DIEP flap	84.61	69.23	
Breast recon with tissue expander	76.92	69.23	
Reduction mammoplasty	69.23	84.61	
Abdominoplasty	46.15	69.23	
Augmentation mammoplasty	84.61	69.23	
Removing skin lesions	46.15	84.61	
Panniculectomy	46.15	69.23	
**Mean**	64.83	73.62	0.272

Abbreviation: AI, artificial intelligence; ASPS, American Society of Plastic Surgeons; DIEP, deep inferior epigastric perforator; recon, reconstruction.

**Table 4:** Mean quality scores by domain assessed by the National Quality Forum

	ASPS (mean score)	AI (mean score)	*p* -Value
Description of procedure	2	2	–
Description of how the procedure will be performed	0.86	1.14	0.60
Rationale for procedure	1.43	2	0.03
Patient-oriented benefits	1.14	1.43	0.69
Quantitative probability	0	0	–
Qualitative probability	1	1	–
Alternatives	2	2	–

Abbreviation: ASPS, American Society of Plastic Surgeons.

## Discussion

Our results indicate that without prompting for a specific grade level, ChatGPT-generated informed consent forms increased the ease by an entire tier of education from “best understood by 4-year university graduates” to “best understood by 2-year college graduates.” The Flesch Reading Ease Scores range from 0 to 100, with 100 being easily understood by an 11-year-old. Informed consents for breast reconstruction with DIEP flap and skin lesion removals had Flesch Reading Ease Scores of 55.57 and 50.32, respectively, which is at the reading level that would be considered difficult to be understood by 13- to 15-year-old students. However, after the conversion, the Flesch–Kincaid Grade level to understand the texts decreased by about three grade levels from post-high school reading levels to mid-high school reading levels.

The ideal length of an informed consent form has been proposed to be less than 1,250 words; beyond this, studies have shown that people are less likely to read the full length of the text. 2 The average word count generated by ChatGPT adhered to the ideal standard, which increases the likelihood of patients reading the forms fully.

Another unique metric in our study related to readability was lexical density, which is calculated by the total number of lexical words, or “content” words, divided by the total number of words. “Content” words are defined by nouns, adjectives, verbs, and adverbs, while other functional words, such as *a, the, and, has* are considered non-lexical. 17 If a text has a higher lexical density, this implies that the text will be lengthier and has an increased level of difficulty. 17 There are general estimates that the lexical density of spoken texts are 40% less than that of written texts. 18 Our analysis shows that post-conversion informed consent texts have lexical densities similar to those of spoken language, contributing to past studies that larger language models can model human vernacular. 15

Quality scores generated by the NQF scale did not differ significantly between the ChatGPT-generated and ASPS-generated consent forms. Additionally, ChatGPT described a condition-specific clinical rationale for the procedure more than ASPS forms.

Taken together, ChatGPT and other large language models may have a role in reforming patient-friendly informed consents used in PRS. Of note, all informed consents produced by both ASPS and ChatGPT failed to provide a quantitative probability for procedure-specific risks, which is one domain of the NQF quality assessment. Non-specific quantification of procedural risks, such as “there is a very low probability of [a risk] occurring,” has been associated with patient skepticism and emotional (both hopeful and fearful) processing of the information. 18 Specific and quantitative risks, “there is a 1–5% probability of [a risk] occurring,” should be verbalized for effective informed consent. Refining the ChatGPT prompts used to generate informed consents can be used as a tool to address this gap. It is important to emphasize that, although AI can improve readability for patients, providers are still responsible for assessing content accuracy in consent forms. There are clinical and legal challenges to the AI generation of these quantitative risks, without verification of clinically grounded research. Together with patient-centered communications, physicians can supplement office visits with these forms to deliver accurate and comprehensible information to support patients' decision-making process.

Our study is not without limitations. We generated informed consents for seven common PRS procedures; however, our results may not be generalizable to informed consents for other PRS and PRS subspecialty procedures. Additionally, we input the prompt “generate a patient-friendly informed consent for [procedure]” rather than specifying a reading level. While our AI-generated texts reduced the reading level compared to the ASPS forms, amending the inputted commands could help achieve the ideal fourth- to sixth-grade reading level. AI was asked to produce a “patient-friendly” document, whereas ASPS forms are forms designed to meet specific legal and administrative requirements. These requirement differences may favor AI-generated texts in NQF quality measures. Lastly, our study did not provide informed consent forms to real providers and patients to rate the quality and readability. Quantitative analysis of information retention, such as whether critical complications were omitted in AI-generated texts, was not performed in the study. Future studies should consider evaluating congruence between readability and quality scores with real provider–patient use and comprehension.

There is a clear gap between established patient-friendly standards and the informed consents used in practice. Large language models have the potential to efficiently adapt informed consents to achieve evolving patient-centeredness standards and improve provider-patient interactions. Our study contributes to previous studies exploring the potential of AI to generate informed consents and highlights how AI can help optimize the shared decision-making process.

### Conclusion

AI, such as ChatGPT, has great potential to improve the readability of PRS-informed consent forms by significantly improving ease with increased conversational content and decreased reading and grade levels while maintaining brevity and accuracy of the intended content. Our results suggest that consent forms can improve the text description of how the procedure is performed, the clinical rationale, patient-oriented benefits, and quantitative assessment of the risks with the use of a readily available AI interface. These are exploratory findings that need to be confirmed in studies with real patients and clinicians. Future studies should be performed to assess patients' understanding of procedures, comparing existing consent forms to those generated at specific literacy levels.
