# Supplementary material for: The Use of Artificial Intelligence to Improve Readability and Quality of Informed Consent Forms in Plastic and Reconstructive Surgery
Source: Arch Plast Surg. 2026 Jul 7;53(5):450–5. doi: 10.1055/a-2870-8035 (PMC13638244; doi:10.1055/a-2870-8035)
Supplement: Supplementary file 1 [file 10-1055-a-2870-8035_29099300.pdf]

Appendix

Modified National Quality Forum Scale

Description of Procedure

1. Is language describing what the procedure is (beyond the medical name) provided for the patient? (yes = 2 points, no = 0)
2. Is a description of how the procedure will be performed provided for the patient? (yes = 2 points, no = 0)

Rational for Procedure

3. Is the clinical rationale (condition-specific justification) for why the procedure will be performed provided? (yes = 2 points, partly =1, no = 0)

Patient-oriented benefit(s)

4. Is any patient-oriented benefit provided (intended impact on patient's health, longevity, and/or quality of life)? (yes = 2 points, no = 0)

Probability of Procedure-specific risks

5. Is a quantitative probability provided for any procedure-specific risk? (yes = 2 points, no = 0)
6. Is a qualitative probability provided for any procedure-specific risk? (yes = 1 point, no = 0)

Alternative(s) to the Procedures

7. Is any alternative provided for the patient? (yes = 2 points, no = 0)
